# Cochlear Implantation in Autoimmune Inner‐Ear Disease: Outcome and Patient‐Reported Benefit

**DOI:** 10.1002/lary.70548

**Published:** 2026-04-07

**Authors:** Merete Hartmann, David Oestreicher, Marc Albert Hüser, Dirk Beutner, Christian Wrobel

**Affiliations:** ^1^ Department of Otorhinolaryngology, Head and Neck Surgery University Medical Center Göttingen Göttingen Germany

**Keywords:** autoimmune‐inner‐ear disease, cochlear implant, sensorineural hearing loss, systemic immune disease, vasculitis

## Abstract

**Objective:**

To investigate cochlear implant (CI) outcomes in patients with hearing loss related to secondary autoimmune inner ear disease (AIED) compared to a matched control cohort.

**Patients and Methods:**

In this retrospective cohort study, we included seven patients with profound sensorineural hearing loss (SNHL) related to secondary AIED treated with a CI. Data on pre‐ and postoperative pure‐tone audiometry, speech perception in quiet and noise (German Freiburg mono‐ and multisyllable test, Oldenburg sentence test), and patient‐reported outcome measures (Speech, Spatial, and Qualities of Hearing Scale, SSQ12) were assessed and compared to a matched control cohort.

**Results:**

Postoperative recognition of monosyllables (65 dB_SPL_) in AIED patients and controls showed significant improvement (*p* < 0.0001) after 12 months with gains of 54 and 58 percentage points, respectively. However, AIED patients required a substantially higher signal to achieve 50%‐word recognition in sentence testing in noise compared to controls. Notably, subjective hearing abilities tested in the SSQ12 in both groups improved substantially, reaching 4.4 [2.4–6.5] in the AIED group and 5.1 [4.5–5.8] in controls at 12 months, showing a convergence of scores compared to the preoperative group difference.

**Conclusion:**

AIED patients substantially benefit from cochlear implantation despite potentially ongoing chronic inner‐ear inflammation. In contrast to poorer performance in classical audiological test settings such as monosyllable presentation and word recognition in noise, self‐perceived hearing was reported nearly as favorable as in the control cohort. Timely diagnosis, tailored preoperative imaging, and individualized rehabilitation are key to improved CI performance in AIED patients.

**Level of Evidence:**

3.

## Graphical abstract

This study demonstrates that patients with secondary autoimmune inner‐ear disease (AIED) achieve significant improvements in speech perception and quality of life following cochlear implantation. Although AIED patients may show slightly lower performance in noisy environments compared to controls, their subjective hearing benefit converges with matched cohorts. Consequently, cochlear implantation remains a highly effective intervention for AIED‐related profound hearing loss despite the underlying chronic inflammation.

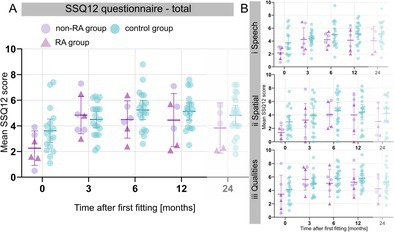

## 1 Introduction

**FIGURE 1:**
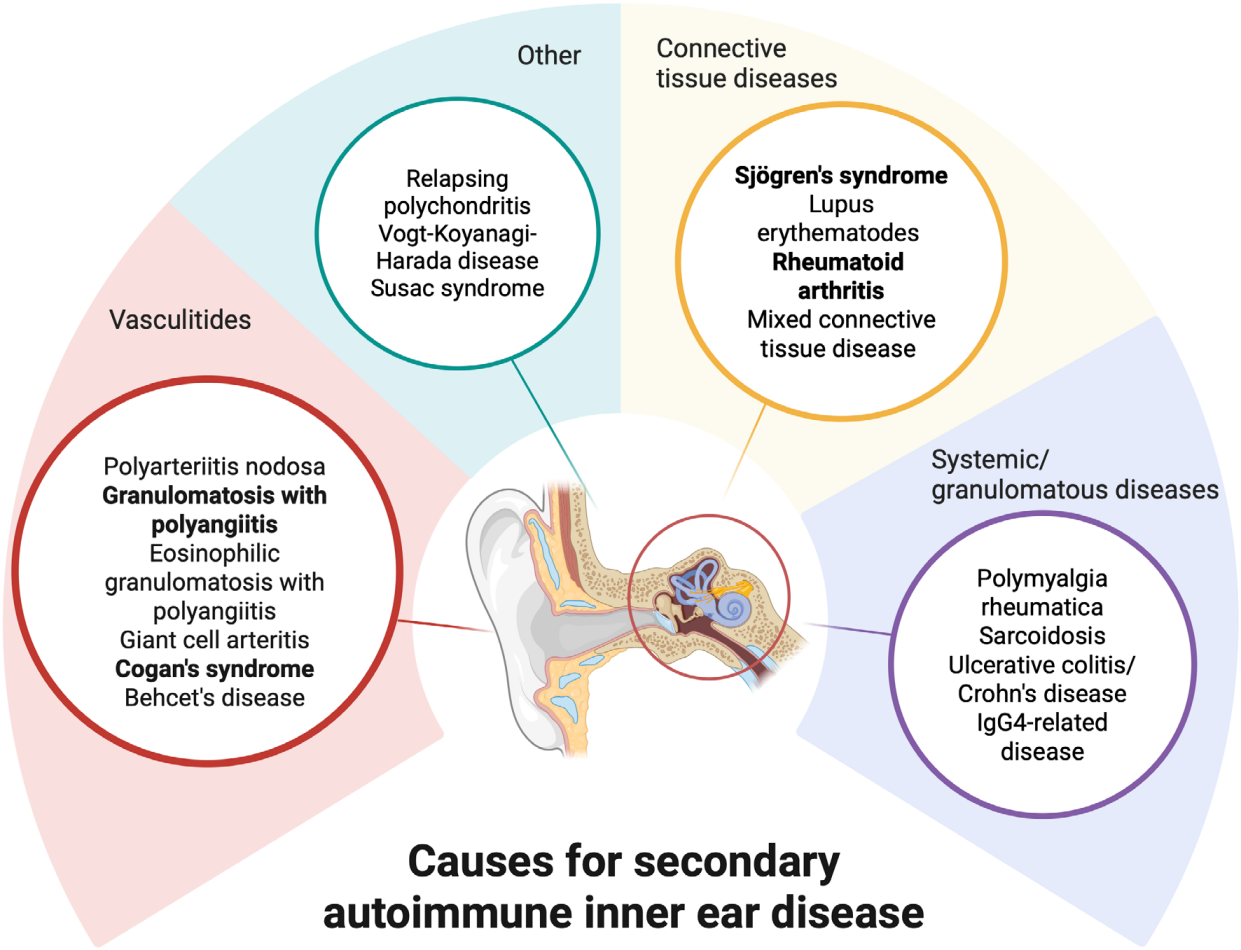
Diseases related to secondary AIED grouped according to their rheumatological classification as vasculitis, connective tissue disease, systemic or granulomatous disease, and other not further classified pathologies. Created in Biorender.com. [Color figure can be viewed in the online issue, which is available at www.laryngoscope.com]

The term “autoimmune inner‐ear disease” (AIED) is referred to as an idiopathic, rapidly progressive, bilateral sensorineural hearing loss (SNHL) that characteristically occurs over a period of weeks or months [1]. Although understanding of systemic autoimmune manifestations has advanced markedly, reflected in updated ACR/EULAR criteria (American College of Rheumatology/European League Against Rheumatism, 2010 and 2022), knowledge of AIED remains limited, and definitive diagnostic criteria are still lacking [2, 3, 4]. AIED may present as an isolated inner‐ear disorder (primary AIED) or in association with systemic autoimmune disease (secondary AIED), the latter accounting for ~15%–30% of cases [5, 6, 7, 8]. Systemic autoimmune conditions associated with AIED can be grouped into four categories (Figure 1): systemic vasculitides (e.g., ANCA‐associated vasculitis), connective‐tissue and systemic autoimmune diseases (e.g., rheumatoid arthritis), granulomatous disorders (e.g., sarcoidosis), and other immune‐mediated syndromes [5, 6, 9, 10]. Epidemiological data for AIED is limited due to the heterogeneity of the associated systemic immune diseases and lack of reproducible diagnostic criteria for the locally confined form of AIED, therefore estimating 5/100,000 cases per year [11]. AIED predominantly affects females and symptoms develop during the third to the sixth decade of life [11]. Although progressive bilateral hearing loss is characteristic for AIED, early unilateral and fluctuating patterns are also reported, often accompanied by tinnitus, vertigo, or aural fullness, which may mimic Ménière's disease [12, 13]. A study looking at audiometric data of 53 AIED patients reported downsloping audiograms for 43% of the cohort, while 21% showed flat or upsloping patterns [14]. Intraoperatively, intracochlear fibrosis as well as ossification is encountered in approximately 54% of AIED patients undergoing cochlear implantation [12]. A systematic review also reported disease‐related intraoperative adjustments in 57.7% of studies, most commonly cochlear drill‐out (53.3%), difficult round‐window insertion (26.7%), and scala‐vestibuli insertion (26.7%) [15].

To date, no reliable serological panel exists for diagnosing AIED. Although candidate markers such as heat shock protein 70 (HSP70) have been proposed, diagnosis still relies on excluding alternative etiologies and assessing corticosteroid responsiveness [16, 17]. High‐dose systemic corticosteroids remain the first‐line treatment, with intratympanic administration reserved for non‐responders [18]. Due to steroid resistance and side effects, disease‐modifying antirheumatic drugs (DMARDs) are increasingly used as a second line therapy [19, 20]. Reviewing 16 studies including 459 patients with AIED, Breslin et al. noted consistent use of cyclophosphamide, methotrexate and azathioprine besides corticosteroids, yet evidence for hearing preservation remains inconsistent. Emerging biologic therapies including TNFα‐inhibitors, IL‐1‐inhibitors and the CD20‐inhibitor rituximab show promising but inconclusive results [19, 20, 21, 22].

Patients unresponsive to first‐ or second‐line therapy often progress to severe‐to‐profound SNHL, consequently, cochlear implantation should be considered. A recent review summarized 29 studies including 115 patients with AIED, demonstrating overall hearing improvement after implantation in both primary and secondary AIED. Based on these findings, early cochlear implant has been recommended to optimize outcomes [23].

Due to the heterogeneity and limited numbers of published cohorts, we investigated hearing outcomes in CI‐patients diagnosed with secondary AIED, assessing speech perception, self‐perceived hearing outcomes relative to age‐ and sex‐matched controls. Consistent with current literature, our findings demonstrate that a CI provides substantial benefit for AIED patients, particularly when diagnosis is made early and cochlear pathology is managed accurately.

## 2 Methods

### 2.1 Patients and Study Design

In this retrospective case–control study, we identified adults who underwent cochlear implantation in cases of secondary AIED at the University Medical Center Göttingen between 2009 and 2024. The study was approved by the institutional ethics committee (No. 13/8/25). Medical records were reviewed to collect demographic, clinical, and audiological data. Only patients with complete preoperative assessments and at least 12 months of postoperative audiological and questionnaire follow‐up were included. Since follow‐up data beyond 12 months were limited due to recent implantations, the 12‐month time point served as primary reference for analysis.

Inclusion criteria comprised bilateral moderate to severe hearing loss or deafness in patients with systemic autoimmune disease, diagnosed according to current rheumatologic guidelines (ACR/EULAR) by a specialized rheumatologist. For each AIED case, the three best matching controls without autoimmune disease implanted within the above defined time window were selected. Best matching was performed hierarchically according to sex, age at cochlear implantation, severity of hearing loss on the implanted side, CI electrode type, and degree of hearing loss on the contralateral side. All patients received unilateral implantation with either a Cochlear (CI522, CI622) or MED‐EL (Synchrony/Synchrony 2) device. Exclusion criteria encompassed re‐implantation, syndromic conditions, learning or motor disabilities, and bilateral sequential implantation. Detailed demographic and implant data, as well as medical history and criteria for diagnosis of autoimmune disease are provided in Tables S1 and S2. All patients received technical fitting and auditory rehabilitation by a specialized team and were monitored regularly postimplantation.

### 2.2 Audiometric Testing and Patient Questionnaires

Audiometric testing was performed in a standard double‐walled, sound‐attenuating booth. Pure‐tone audiometry (PTA, DIN EN ISO 8253‐1) and speech audiometry, including word and sentence recognition (DIN EN ISO 8253‐2), were performed using a calibrated AT1000 audiometer (AURITEC GmbH, Hamburg, Germany; EN 60645) according to clinical standards. The German Freiburg speech test (multisyllabic numbers and monosyllabic words) was administered preoperatively with and without best‐aided devices and at each postoperative follow‐up at 55, 65, 80 dB sound pressure level (SPL). Sentence recognition was assessed from 3 months post‐activation using the Oldenburg sentence test (OLSA) in quiet and in noise. All postoperative speech perception measurements were performed in a CI‐only condition with contralateral hearing attenuation. The short form (SSQ12) of the Speech, Spatial Qualities of Hearing Scale was used to assess self‐perceived hearing disability, covering speech perception, spatial hearing, and sound quality. The SSQ12 demonstrates strong correlation with the full SSQ, a validated instrument widely used in CI research [24, 25, 26]. Its brevity enhances feasibility and patient compliance, supporting its use in clinical and research settings.

### 2.3 Statistical Analysis

Due to the limited sample size, the analysis emphasized descriptive comparison. Statistical evaluation was conducted using GraphPad Prism (GraphPad Software, San Diego, CA, USA). Data normality was assessed via the Shapiro–Wilk test. Group differences were evaluated using two‐sided unpaired *t*‐tests for normally distributed data and Wilcoxon rank‐sum tests for non‐normal distributions. Statistical significance was defined as *α* = 0.05 (*).

## 3 Results

**FIGURE 2:**
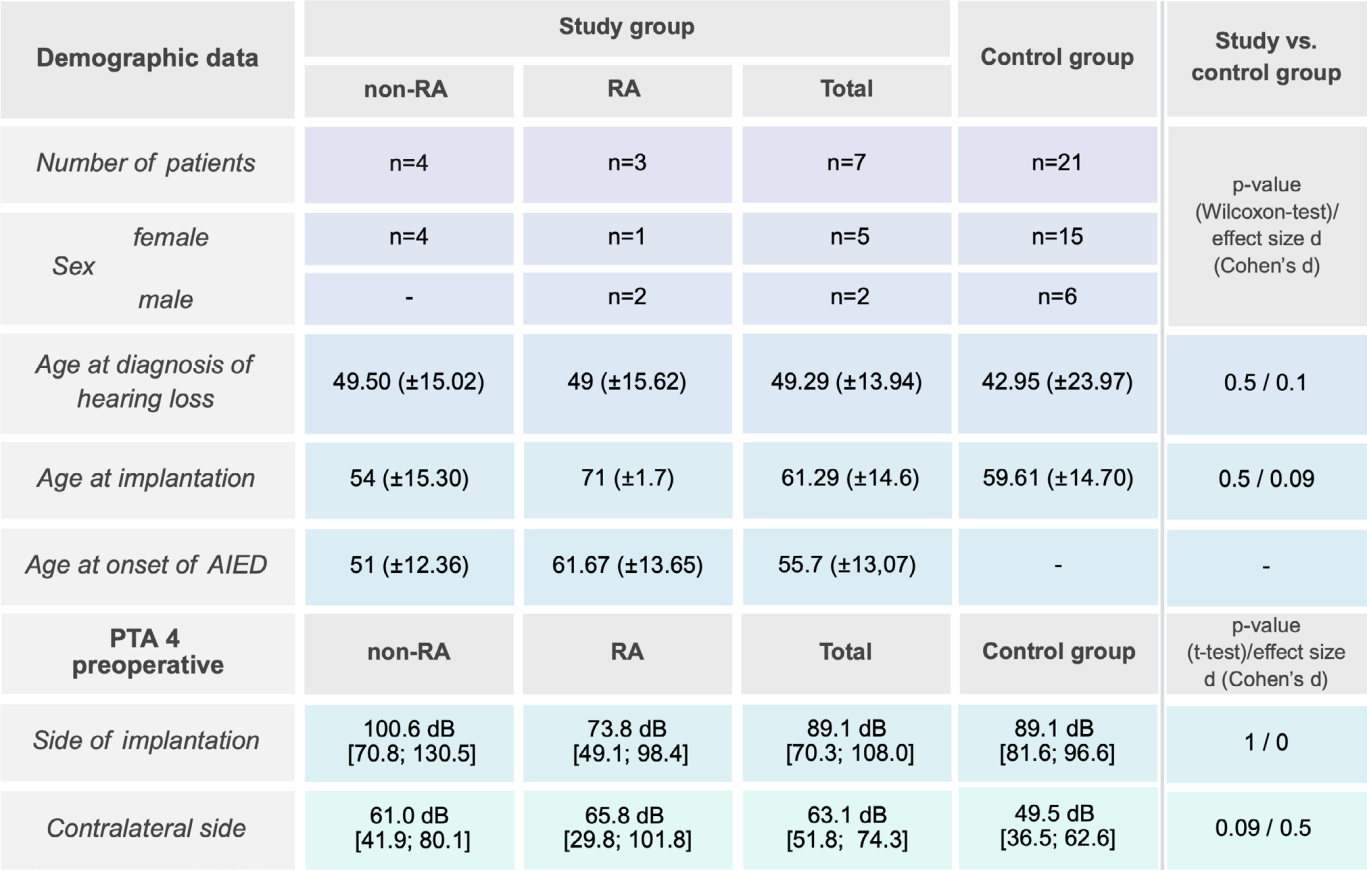
Demographic and audiometric characteristics of the study cohort, non‐RA, RA subgroup and control group. PTA4 (pure tone average) of 0.5, 1, 2, and 4 kHz; PTA4 values are mean ± 95% confidence interval; age is mean ± SD (standard deviation). Created in Biorender.com.

**FIGURE 3:**
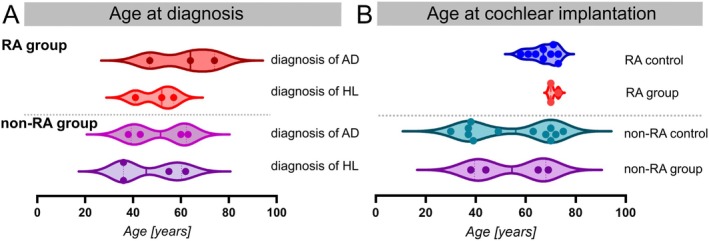
(A) Violin plots display the age at onset of hearing loss and diagnosis of autoimmune disease in the AIED cohort, shown separately for patients with rheumatoid arthritis (RA) and non‐RA etiologies. (B) Age at cochlear implantation is similarly compared between AIED subgroups and corresponding controls. [Color figure can be viewed in the online issue, which is available at www.laryngoscope.com]

Covering a 15‐year period (2009–2024), we identified seven patients with secondary AIED who met the inclusion criteria for this study and selected 21 matched controls. Baseline and disease specific characteristics of both groups are listed in Figure 2. Regarding the selection criteria for the control group, differences compared to the study cohort were considered minimal and clinically negligible. Further details on demographic and implant data are provided in Table S1. Figure 2 provides statistical analyses supporting this assumption; however, given the small number of AIED patients, the validity here is limited. Two AIED patients experienced onset of hearing loss before age 40 (Figure 3A) and were implanted earlier, while RA patients underwent implantation mainly after age 60, a pattern mirrored in the matched controls (Figure 3B). Given the older implantation age and distinct systemic disease profile of RA, the AIED group was further subdivided to account for potential age‐ and disease‐related differences.

In our cohort one patient was diagnosed with Cogan's syndrome after presenting with fluctuating hearing loss, tinnitus, vertigo, and interstitial keratitis. Treatment required systemic prednisolone with escalation to methotrexate and later infliximab due to disease progression with acute aortitis. A second patient developed sudden progressive hearing loss with vertigo and was diagnosed with Sjögren's syndrome based on typical sicca symptoms, subsequently receiving long‐term low‐dose prednisolone. Two patients were diagnosed with ANCA‐associated vasculitis, one of them, suffering from granulomatosis with polyangiitis (GPA), experienced unilateral acute hearing loss during a disease flare and was escalated from prednisolone to rituximab upon progression of pulmonary disease. Another patient with microscopic polyangiitis developed progressive hearing loss, tinnitus and vertigo was treated with prednisolone followed by azathioprine. Three patients were diagnosed with RA, two of them were seropositive for rheumatoid factor and anti‐cyclic citrullinated peptide antibodies. Two were treated with leflunomide, with one transitioning to etanercept due to progression and later treatment with methotrexate and adalimumab; the third seronegative RA patient received recurrent prednisolone before initiation of methotrexate. A detailed summary of the patient's medical history and treatment can be found in Tables S2 and S3.

Cochlear implantation was performed by highly experienced surgeons in all patients. Preoperative evaluation and surgical planning comprised radiological imaging, comprehensive audiological and vestibular assessment, interdisciplinary case discussion within our CI board, and detailed patient counseling. As anticipated from preoperative imaging, one patient with vasculitis exhibited round window ossification intraoperatively, necessitating a cochleostomy to achieve complete electrode insertion. In another case of vasculitis, cochleostomy combined with intracochlear dilation using a dummy electrode was required to overcome fibrosis and enable full insertion of the electrode array. In the latter case postoperative CT imaging confirmed complete insertion, though with bone compaction and mildly uneven positioning. No further intraoperative complications were observed.

**FIGURE 4:**
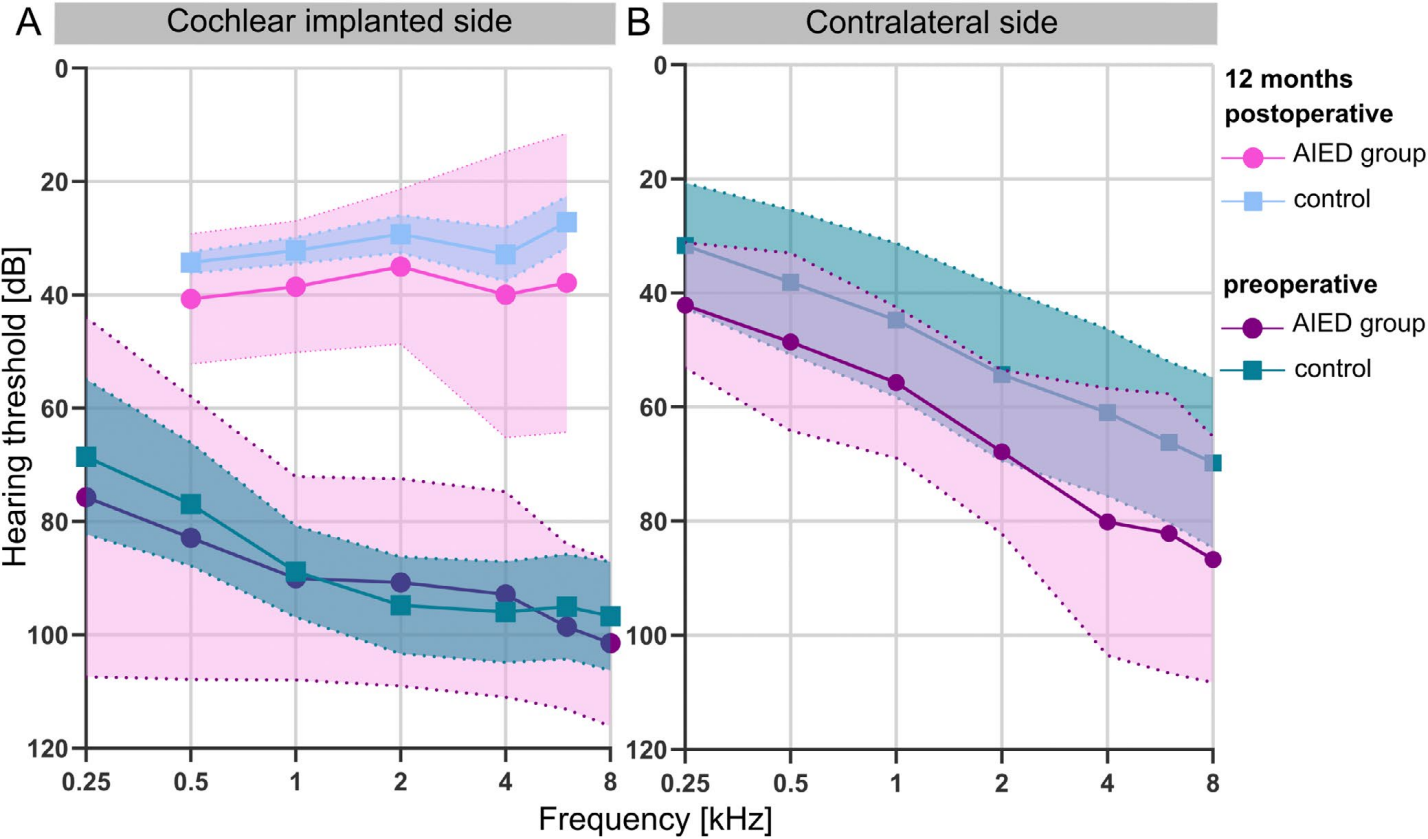
Averaged pure‐tone audiometry results for the AIED (purple) and control (turquoise) groups are shown preimplantation for the implanted ear (A) and contralateral ear (B). (A) Also includes cumulative thresholds 12 months post‐CI for AIED (pink) and controls (light blue). Data are presented as means with 95% confidence intervals (shaded area). [Color figure can be viewed in the online issue, which is available at www.laryngoscope.com]

First, patient outcomes following cochlear implantation were evaluated by pre‐ and postoperative PTA (Figure 4). Prior to surgery, both groups showed comparable results across all frequencies on the implanted and the contralateral side. CI‐aided PTA‐measurements in an open field setting 12 months after surgery (Figure 4, upper left slopes) indicated a mild tendency toward poorer performance in the AIED group compared to the controls. Furthermore, the CI‐driven hearing thresholds in these patients varied considerably.

**FIGURE 5:**
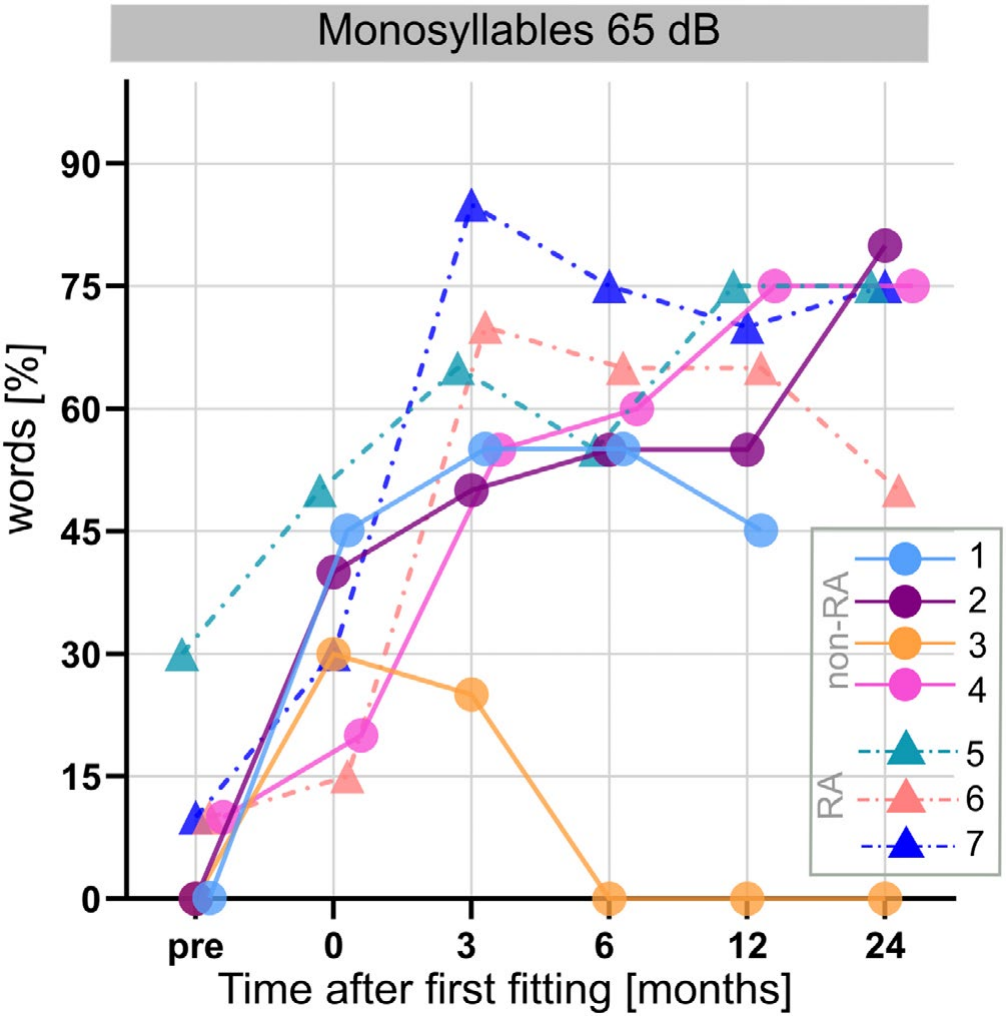
Monosyllable perception at 65 dB_SPL_ for individual AIED patients (Nos. 1–7) over 24 months of follow‐up. Non‐RA patients are shown as colored circles (Nos. 1–4) and RA patients as dotted lines with rectangles (Nos. 5–7). Data points are slightly offset for clarity. [Color figure can be viewed in the online issue, which is available at www.laryngoscope.com]

Postoperative speech perception in AIED patients, assessed individually over time, reached acceptable levels overall. Six out of seven patients showed improved speech comprehension to over 55% word‐recognition (monosyllables presented at 65 dB_SPL_) within 6 months; four exceeded 75% by 24 months (Figure 5). From the 6‐month assessment onward, one AIED patient could not obtain usable auditory information from electrical cochlear stimulation below the pain threshold and ceased using the implant. This patient, diagnosed with GPA, also demonstrated pronounced intraoperative fibrosis and intracochlear ossification. This patient was excluded from further analysis due to incomplete follow‐up data, particularly at the 12‐month endpoint. The case, however, is discussed in detail given its clinical relevance.

**FIGURE 6:**
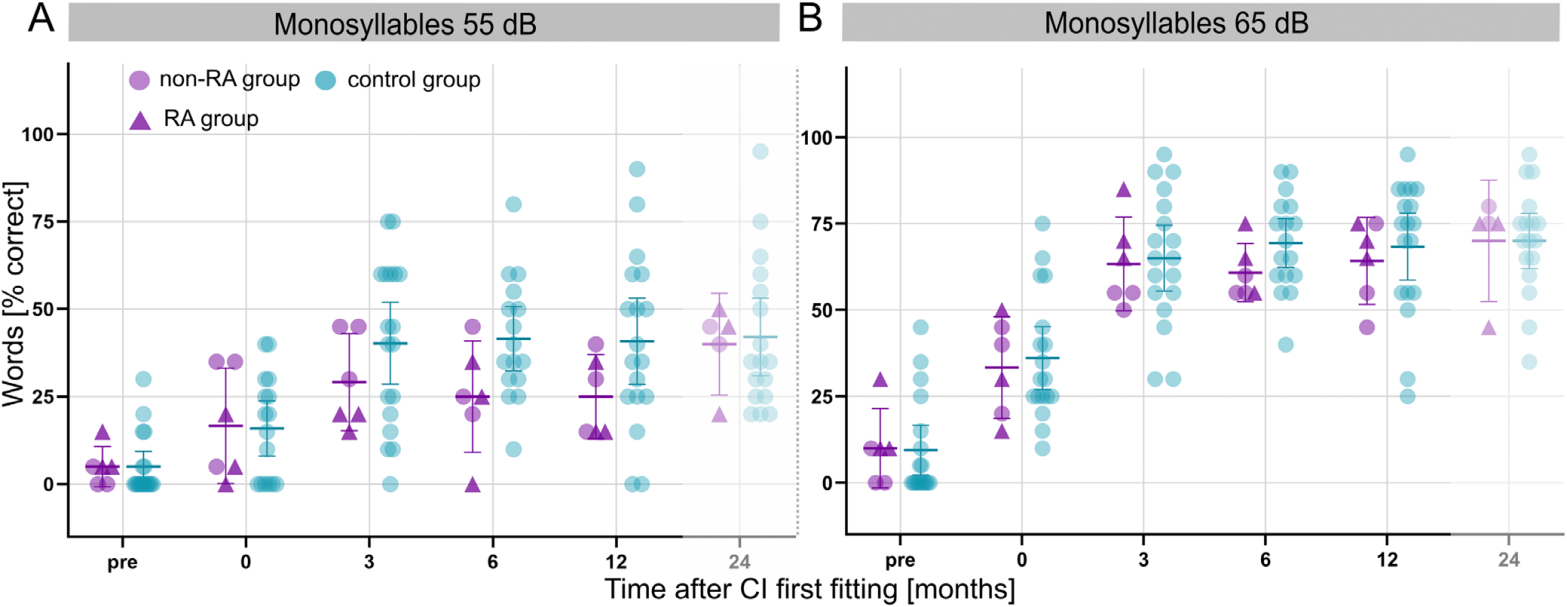
Cumulative monosyllable recognition (Freiburg test) at 55 (A) and 65 dB_SPL_ (B) across pre‐ and postoperative follow‐ups. AIED patients are shown in purple (non‐RA: Circles; RA: rectangles) and controls in turquoise. Individual data points are plotted with mean values and 95% confidence intervals. Data at 24 months are shown transparent owing to reduced patient data availability at this timepoint, same in Figures 7 and 8. [Color figure can be viewed in the online issue, which is available at www.laryngoscope.com]

To assess word recognition, monosyllables were presented at 55 and 65 dB_SPL_. At 65 dB_SPL_, the standard follow‐up level, both groups showed marked improvement 12 months after cochlear implantation compared to preoperative scores, with gains of 54 and 58 percentage points in the AIED group and controls, respectively (*p* < 0.0001 for both groups). Despite similar performance at 65 dB_SPL_, the AIED group showed a smaller benefit at 55 dB_SPL_, improving by 20 compared to 35 percentage points in controls. Across the 3‐, 6‐, and 12‐month follow‐ups, mean recognition at 55 dB_SPL_ remained consistently lower in the AIED group (Figure 6). Although the AIED group improved at 55 dB_SPL_ over 12 months, this did not reach statistical significance (*p* = 0.09), likely influenced by the limited group size. Figure S1 reports the audiometric data according to the American Academy of Otolaryngology—Head and Neck Surgery minimal reporting standard.

**FIGURE 7:**
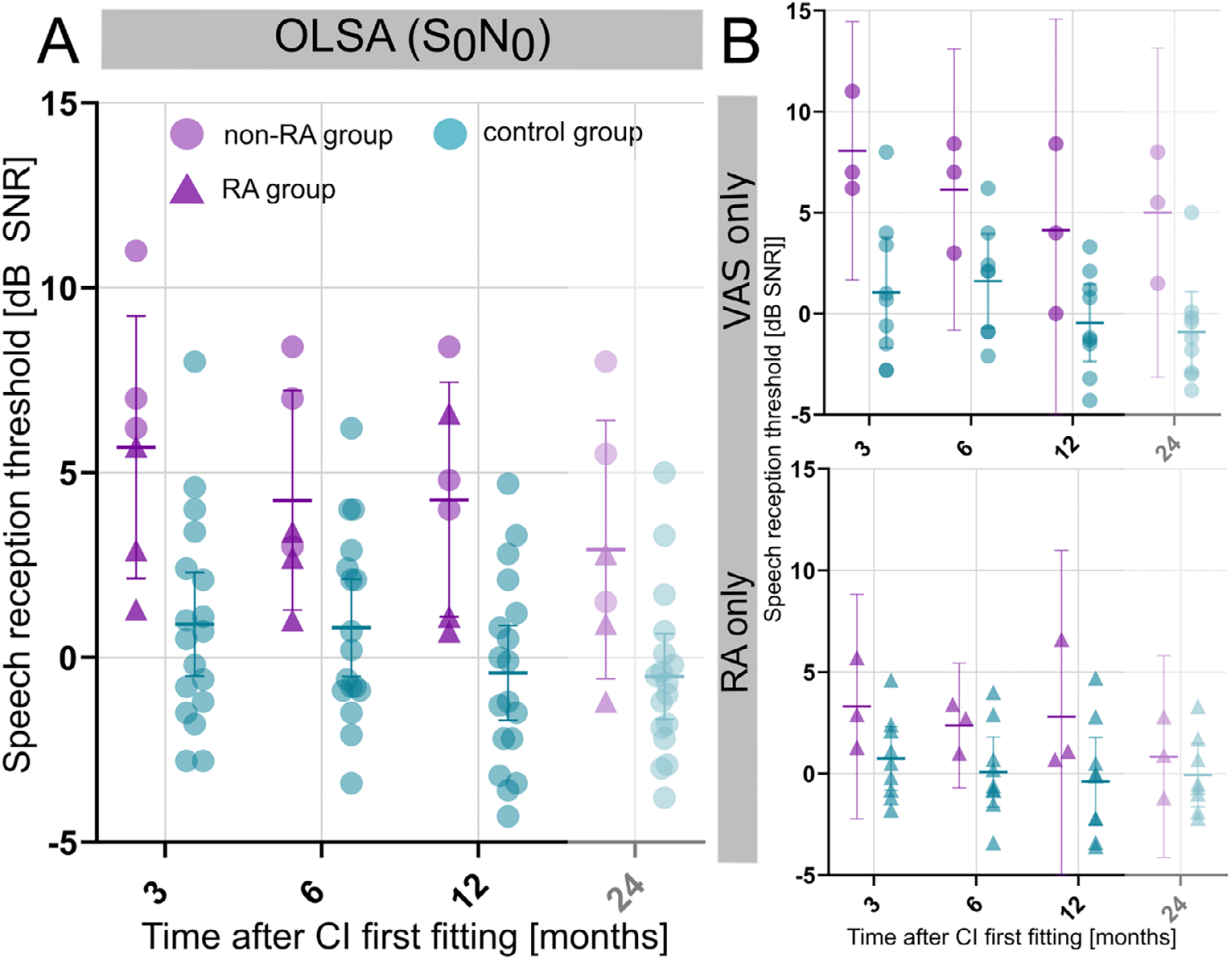
(A) Postoperative results of sentence‐in‐noise testing (OLSA) for AIED (purple; non‐RA: circles, RA: rectangles) and controls (turquoise), shown as individual data points with mean and 95% confidence intervals. (B) Values displayed separately for non‐RA and RA subgroups and their respective controls. [Color figure can be viewed in the online issue, which is available at www.laryngoscope.com]

Patients' speech reception threshold (SRT) was assessed using the OLSA test in noise at 65 dB_SPL_. Both groups showed progressive improvement over the follow‐up period up to 12 months (Figure 7). Across all time points, AIED patients required a substantially higher signal‐to‐noise ratio compared to the control group to achieve 50%‐word recognition. This effect was particularly pronounced in non‐RA patients (Figure 7B), with a SNR of 4.1 dB [−6.3; 14.5 dB] compared to SNR of the control group of −0.4 dB [−2.3; 1.4 dB] and the RA group with SNR of 2.8 dB [−5.3; 10.9 dB] after 12 months [lower and upper 95% confidence interval].

**FIGURE 8:**
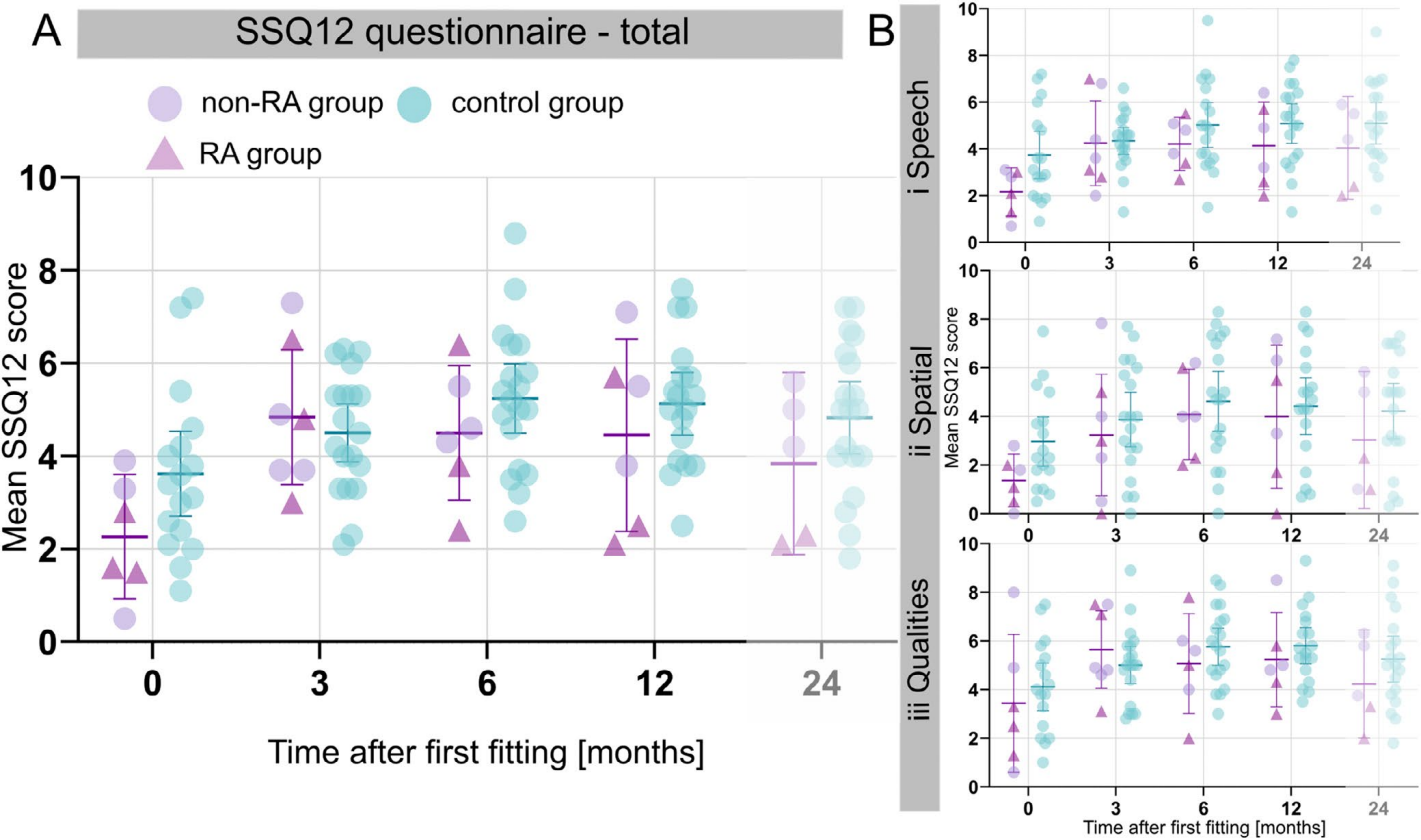
Postoperative SSQ12 self‐assessment results are shown over time for total scores (A) and for subscales (B) speech (i), spatial (ii), and qualities (iii). The AIED group is shown in purple (non‐RA: circles; RA: rectangles) and controls in turquoise. Individual scores are plotted with means and 95% confidence intervals. [Color figure can be viewed in the online issue, which is available at www.laryngoscope.com]

Self‐perceived hearing function, as assessed by the SSQ12 questionnaire, was largely comparable between the AIED group and the control population throughout follow‐up. At baseline, patients with AIED reported notable lower scores (2.2 [0.9; 3.6]) than controls (3.6 [2.7; 4.5]), despite being matched according to preoperative audiological performance (Figure 8). During the first year after implantation, subjective hearing abilities in both groups improved substantially, reaching 4.4 [2.4; 6.5] in the AIED group and 5.1 [4.5; 5.8] in controls at 12 months, showing a convergence of scores after cochlear implantation.

## 4 Discussion

This study highlights three key observations: First, despite the underlying autoimmune‐mediated chronic inflammation of the inner ear, patients' hearing benefited substantially from cochlear implantation. Second, there was a trend toward poorer performance in audiological test settings for AIED patients, particularly for monosyllable recognition at lower presentation levels (55 dB_SPL_) and word recognition in noise. Third, in contrast to these findings, self‐perceived hearing (SSQ12) improved to levels comparable to other CI recipients, indicating that everyday hearing with the implant is nearly as favorable as in non‐AIED patients.

These results align with previous controlled cohorts: post‐implant speech outcomes in AIED can approach those of non‐AIED CI users [13, 23, 27]. Aftab et al. reported comparable short‐ and long‐term word and sentence scores, with excellent average performance in both groups [27]. In a larger comparative cohort, Wang et al. observed high open‐set sentence scores without disadvantage relative to matched controls [10]. The present data echo those experiences with post‐implant gains at 6–12 months in monosyllable recognition at 65 dB_SPL_ for most implant recipients. Notably, our observation of poorer speech‐in‐noise performance in AIED patients compared with corresponding controls nuances the “on‐par with controls” narrative that is often based on quiet listening tests. Most earlier studies emphasized monosyllable and open‐set sentence scores in quiet; few directly assessed SRT in noise.

Patient‐reported hearing improved markedly after CI and largely converged between groups: although AIED patients started with lower SSQ12 scores, both cohorts improved over the first year, with similar trends across all subscales. Consistent with the literature, the SSQ12 questionnaire captures real‐world communication that improves substantially after cochlear implantation. However, patient‐reported outcome measures (PROMs) correlate only modestly with clinic speech tests, as PROMs reflect aspects such as listening effort and situational coping beyond recognition scores alone [24, 25, 28, 29].

Our key findings indicate that while cochlear implantation restores hearing in AIED, residual deficits—especially in noise—may persist, plausibly reflecting ongoing or irreversible cochlear as well as neural injury attributable to the underlying immune process [19, 30, 31]. While it is tempting to assume that cognitive and auditory integration deficits coexist in CI recipients with AIED, further complicating auditory rehabilitation, there is currently a lack of studies to support this hypothesis.

In our cohort, one patient presented with round window ossification and another with cochlear fibrosis, both of which necessitated a cochleostomy. In each case, preoperative imaging revealed radiological findings suggestive of the respective pathology. These findings were therefore anticipated preoperatively, incorporated into surgical planning, and thoroughly discussed with the patient during preoperative counseling. A substantial proportion (50%–54%) of AIED patients demonstrate intracochlear fibrosis and ossification at implantation showing tendency but no significant evidence that perioperative complication rates exceed those seen in CI candidates without AIED [12, 13, 23, 27]. These data support early CI referral once pharmacotherapy is no longer effective, emphasize the need for preoperative advanced imaging to detect cochlear ossification, and underscore the importance of targeted briefing of the surgical team and patient counseling about the potential need for more invasive surgical approaches.

Although most AIED patients reached clinically meaningful outcomes by 6–12 months and some improved further, a minority showed deterioration over time, including one case with no usable auditory benefit despite complete insertion. Similar idiosyncratic courses have been described, including cases of performance decline in suspected Cogan's syndrome [10, 12, 23, 32]. Potential contributors include ongoing immune‐mediated cochlear damage, fibrosis affecting electrode‐neuron interfaces, and limited central adaptation. Although definitive mechanisms remain unclear, these findings highlight the importance of long‐term monitoring, timely troubleshooting—including imaging and re‐mapping—and close coordination with rheumatology for disease control.

### 4.1 Strengths and Limitations

Strengths include a rigorously matched control cohort, standardized outcome measures (monosyllables at 55/65 dB_SPL_ and OLSA SRT in noise), and AIED subgroup analyses. Detailed clinical characterization of patients further enhances internal validity. Limitations relate to the small sample size (7 AIED patients, 21 matched controls) inherent to this rare condition, which increases the risk of type II errors and limits detection of subtle associations. Reduced numbers at the 24‐month time point, heterogeneity of underlying autoimmune diagnoses (vasculitides vs. rheumatoid arthritis), device heterogeneity, and the observational study design further limit causal inference. These constraints mirror the largely retrospective literature; nonetheless, the overall convergence of our results with prior work strengthens external validity.

### 4.2 Conclusion

Accurate and early diagnosis of AIED remains a major challenge, as the disease is frequently overlooked despite characteristic audiological and systemic features. The diagnostic delay (6.4 years after the onset of hearing loss in our study) is likely one of the most decisive obstacles for effective management, since it determines the window for therapeutic intervention and the timing of cochlear implantation. For steroid‐refractory or medically exhausted cases, a CI should be offered as a timely option, with a high likelihood of substantial benefit. In our AIED cohort, hearing loss consistently preceded the diagnosis of the underlying systemic autoimmune disease. This observation suggests that bilateral, often asymmetric SNHL may represent an early clinical manifestation of systemic autoimmune pathology, particularly in cases where the definitive diagnosis has not yet been established and SNHL is accompanied by tinnitus, vertigo, or fluctuating symptoms. However, evidence from the existing literature supporting this temporal association remains limited [33].

PROMs indicate that everyday hearing of AIED patients can approach that of other CI recipients, underscoring the real‐world value of, for example, the SSQ12 even when speech‐in‐noise deficits persist. Counseling should address increased difficulty in noise and especially the risk of cochlear fibrosis or ossification, which can result in limited to no auditory benefit in the worst case. Rehabilitation should prioritize listening‐in‐noise management strategies (e.g., directional microphones, adaptive noise reduction, remote microphone systems), targeted speech‐in‐noise auditory training and routine PROM use to guide individualized care. Although comparative evidence in AIED remains limited, systematic reviews and expert guidance in immune‐mediated SNHL support individualized, multimodal rehabilitation [12, 13, 23].

Future work requires prospective multicenter studies with harmonized speech‐in‐noise metrics, PROMs, and systematic autoimmune phenotyping to refine prognostication and rehabilitation approaches.

## Funding

The authors have nothing to report.

## Conflicts of Interest

The authors declare no conflicts of interest.

## Supporting information


**Table S1:** Patient demographic and clinical data including age, age at onset of hearing loss and implantation, cause of hearing loss, underlying autoimmune disease, side of implantation, type of CI device, and electrode type.
**Table S2:** Detailed clinical data of the study group, including autoimmune disease, serological testing, and organ involvement as well as treatment details.
**Table S3:** Summary of data on drug‐induced ototoxicity of immunosuppressant and immunomodulatory monoclonal antibodies and references discussing cases of hearing loss after treatment.
**Figure S1:** Scattergrams according to the American Academy of Otolaryngology—Head and Neck Surgery minimal reporting standard for reporting audiometric data. Generated using the software “R” (version 4.5.2, R Foundation for Statistical Computing, Vienna, Austria) and the R package from the following publication: Pan DW, Oghalai JS. The AudioScatter Package in RStudio to Generate Scattergrams for Reporting Hearing Results. Otol Neurotol. 2026;47(1):7‐10. doi:10.1097/MAO.0000000000004693

## Data Availability

The data that support the findings of this study are available from the corresponding author upon reasonable request.

## References

[1] B. F. McCabe , “Autoimmune Sensorineural Hearing Loss,” Annals of Otology, Rhinology, and Laryngology 88, no. 5 Pt 1 (1979): 585–589, 10.1177/000348947908800501. 496191

[2] D. Aletaha , T. Neogi , A. J. Silman , et al., “2010 Rheumatoid Arthritis Classification Criteria: An American College of Rheumatology/European League Against Rheumatism Collaborative Initiative,” Annals of the Rheumatic Diseases 69, no. 9 (2010): 1580–1588, 10.1136/ard.2010.138461. 20699241

[3] P. C. Grayson , C. Ponte , R. Suppiah , et al., “2022 American College of Rheumatology/European Alliance of Associations for Rheumatology Classification Criteria for Eosinophilic Granulomatosis With Polyangiitis,” Arthritis & Rhematology 74, no. 3 (2022): 386–392, 10.1002/art.41982. 35106968

[4] J. C. Robson , P. C. Grayson , C. Ponte , et al., “2022 American College of Rheumatology/European Alliance of Associations for Rheumatology Classification Criteria for Granulomatosis With Polyangiitis,” Arthritis & Rhematology 74, no. 3 (2022): 393–399, 10.1002/art.41986. 35106964

[5] A. Ciorba , V. Corazzi , C. Bianchini , et al., “Autoimmune Inner Ear Disease (AIED): A Diagnostic Challenge,” International Journal of Immunopathology and Pharmacology 32 (2018): 2058738418808680, 10.1177/2058738418808680. 30376736 PMC6213300

[6] T. Mijovic , A. Zeitouni , and I. Colmegna , “Autoimmune Sensorineural Hearing Loss: The Otology‐Rheumatology Interface,” Rheumatology 52, no. 5 (2013): 780–789, 10.1093/rheumatology/ket009. 23436581

[7] G. B. Hughes , S. E. Kinney , B. P. Barna , and L. H. Calabrese , “Practical Versus Theoretical Management of Autoimmune Inner Ear Disease,” Laryngoscope 94, no. 6 (1984): 758–767, 10.1288/00005537-198406000-00006. 6374341

[8] T. Arunthanachaikul , S. Osothsinlp , S. Nivatwongs , and P. Narongroeknawin , “Prevalence and Intriguing Clinical Profiles of Autoimmune Inner Ear Diseases in Sudden Sensorineural Hearing Loss,” Otology & Neurotology 46, no. 2 (2025): 215–220, 10.1097/MAO.0000000000004391. 39663802

[9] M. Ralli , V. D'Aguanno , A. Di Stadio , et al., “Audiovestibular Symptoms in Systemic Autoimmune Diseases,” Journal of Immunology Research 2018 (2018): 5798103, 10.1155/2018/5798103. 30211232 PMC6120292

[10] J. R. Wang , H. W. Yuen , D. B. Shipp , et al., “Cochlear Implantation in Patients With Autoimmune Inner Ear Disease Including Cogan Syndrome: A Comparison With Age‐ and Sex‐Matched Controls,” Laryngoscope 120, no. 12 (2010): 2478–2483, 10.1002/lary.21060. 21082747

[11] D. L. George and S. Pradhan , “Idiopathic Sensorineural Hearing Disorders in Adults—A Pragmatic Approach,” Nature Reviews Rheumatology 5, no. 9 (2009): 505–512, 10.1038/nrrheum.2009.150. 19652649

[12] X. Goh , J. Muzaffar , and M. Bance , “Cochlear Implantation in Systemic Autoimmune Disease,” Current Opinion in Otolaryngology & Head and Neck Surgery 30, no. 5 (2022): 291–297, 10.1097/MOO.0000000000000839. 36004773

[13] M. U. Malik , V. Pandian , H. Masood , et al., “Spectrum of Immune‐Mediated Inner Ear Disease and Cochlear Implant Results,” Laryngoscope 122, no. 11 (2012): 2557–2562, 10.1002/lary.23604. 22991211

[14] G. Psillas , G. G. Dimas , M. Daniilidis , P. Binos , T. Tegos , and J. Constantinidis , “Audiological Patterns in Patients With Autoimmune Hearing Loss,” Audiology and Neurotology 27, no. 4 (2021): 336–346, 10.1159/000518694. 34518471

[15] N. Deshpande , N. Aminpour , H. Cheng , J. D. Johns , and M. Hoa , “Cochlear Implantation and Perioperative Management in Autoimmune Inner Ear Disease: A Systematic Review and Meta‐Analysis,” Otology & Neurotology Open 1, no. 2 (2021): e006, 10.1097/ONO.0000000000000006. 38550355 PMC10969513

[16] C. Ianuale , G. Cadoni , E. De Feo , et al., “A Systematic Review and Meta‐Analysis of the Diagnostic Accuracy of Anti‐Heat Shock Protein 70 Antibodies for the Detection of Autoimmune Hearing Loss,” Otology & Neurotology 34, no. 2 (2013): 214–219, 10.1097/MAO.0b013e31827d0b8b. 23295728

[17] K. Yeom , J. Gray , T. S. Nair , et al., “Antibodies to HSP‐70 in Normal Donors and Autoimmune Hearing Loss Patients,” Laryngoscope 113, no. 10 (2003): 1770–1776, 10.1097/00005537-200310000-00020. 14520104

[18] A. J. Matsuoka and J. P. Harris , “Autoimmune Inner Ear Disease: A Retrospective Review of Forty‐Seven Patients,” Audiology and Neurotology 18, no. 4 (2013): 228–239, 10.1159/000351289. 23817208

[19] N. K. Breslin , V. V. Varadarajan , E. S. Sobel , and R. S. Haberman , “Autoimmune Inner Ear Disease: A Systematic Review of Management,” Laryngoscope Investigative Otolaryngology 5, no. 6 (2020): 1217–1226, 10.1002/lio2.508. 33364414 PMC7752060

[20] T. M. Gordis , S. R. Shah , C. Ward , and H. G. Rizk , “Disease‐Modifying Antirheumatic Drugs in the Treatment of Autoimmune Inner Ear Disease: A Systematic Review and Meta‐Analysis of Auditory and Vestibular Outcomes,” Otology & Neurotology 44, no. 1 (2023): 2–9, 10.1097/MAO.0000000000003743. 36509432

[21] B. Balouch , R. Meehan , A. Suresh , et al., “Use of Biologics for Treatment of Autoimmune Inner Ear Disease,” American Journal of Otolaryngology 43, no. 5 (2022): 103576, 10.1016/j.amjoto.2022.103576. 35963108

[22] H. Sakano and J. P. Harris , “Emerging Options in Immune‐Mediated Hearing Loss,” Laryngoscope Investigative Otolaryngology 4, no. 1 (2018): 102–108, 10.1002/lio2.205. 30828626 PMC6383306

[23] J. Lee , K. Biggs , J. Muzaffar , M. Bance , and P. Monksfield , “Hearing Loss in Inner Ear and Systemic Autoimmune Disease: A Systematic Review of Post‐Cochlear Implantation Outcomes,” Laryngoscope Investigative Otolaryngology 6, no. 3 (2021): 469–487, 10.1002/lio2.563. 34195369 PMC8223457

[24] S. Gatehouse and W. Noble , “The Speech, Spatial and Qualities of Hearing Scale (SSQ),” International Journal of Audiology 43, no. 2 (2004): 85–99, 10.1080/14992020400050014. 15035561 PMC5593096

[25] W. Noble , N. S. Jensen , G. Naylor , N. Bhullar , and M. A. Akeroyd , “A Short Form of the Speech, Spatial and Qualities of Hearing Scale Suitable for Clinical Use: The SSQ12,” International Journal of Audiology 52, no. 6 (2013): 409–412, 10.3109/14992027.2013.781278. 23651462 PMC3864780

[26] J. Wyss , D. J. Mecklenburg , and P. L. Graham , “Self‐Assessment of Daily Hearing Function for Implant Recipients: A Comparison of Mean Total Scores for the Speech Spatial Qualities of Hearing Scale (SSQ49) With the SSQ12,” Cochlear Implants International 21, no. 3 (2020): 167–178, 10.1080/14670100.2019.1707993. 31887255

[27] S. Aftab , M. T. Semaan , G. S. Murray , and C. A. Megerian , “Cochlear Implantation Outcomes in Patients With Autoimmune and Immune‐Mediated Inner Ear Disease,” Otology & Neurotology 31, no. 8 (2010): 1337–1342, 10.1097/MAO.0b013e3181f0c699. 20729775

[28] T. McRackan , M. Bauschard , J. Hatch , et al., “Meta‐Analysis of Cochlear Implantation Outcomes Evaluated With General Health‐Related Patient‐Reported Outcome Measures,” Otology & Neurotology 39, no. 1 (2018): 29–36, 10.1097/MAO.0000000000001620. 29227446 PMC5728184

[29] D. J. Mecklenburg , P. L. Graham , and C. J. James , “Relationships Between Speech, Spatial and Qualities of Hearing Short Form SSQ12 Item Scores and Their Use in Guiding Rehabilitation for Cochlear Implant Recipients,” Trends in Hearing 28 (2024): 23312165231224643, 10.1177/23312165231224643. 38361477 PMC10874150

[30] J. A. Brant , S. J. Eliades , and M. J. Ruckenstein , “Systematic Review of Treatments for Autoimmune Inner Ear Disease,” Otology & Neurotology 36, no. 10 (2015): 1585–1592, 10.1097/MAO.0000000000000875. 26485595

[31] M. Camard , F. Urbain , and N. Noel , “Cognitive Impairment in Systemic Autoimmune and Inflammatory Diseases: A Narrative Review Focused on ANCA‐Associated Vasculitis, Sarcoidosis, Sjögren's Syndrome, Systemic Sclerosis, and Behçet's Disease,” Autoimmunity Reviews 24 (2025): 103899, 10.1016/j.autrev.2025.103899. 40769406

[32] A. R. Marrero‐Gonzalez , C. Ward , S. A. Nguyen , S. S. Jeong , and H. G. Rizk , “Audiovestibular Outcomes in Adult Patients With Cogan Syndrome: A Systematic Review,” European Archives of Oto‐Rhino‐Laryngology 282, no. 1 (2025): 23–35, 10.1007/s00405-024-08878-5. 39110231 PMC11735566

[33] S. S. Broughton , W. E. Meyerhoff , and S. B. Cohen , “Immune‐Mediated Inner Ear Disease: 10‐Year Experience,” Seminars in Arthritis and Rheumatism 34, no. 2 (2004): 544–548, 10.1016/j.semarthrit.2004.07.001. 15505770

