# Supplementary material for: Cochlear Implantation in Autoimmune Inner‐Ear Disease: Outcome and Patient‐Reported Benefit
Source: Laryngoscope. 2026 Apr 7;136(9):3980–9. doi: 10.1002/lary.70548 (PMC13460887; doi:10.1002/lary.70548)
Supplement: Supplementary file 1 [file LARY-136-3980-s001.docx]

Supplementary Material

Table S1: patient demographic and clinical data including age, age at onset of hearing loss and implantation, cause of hearing loss, underlying autoimmune disease, side of implantation and CI device and electrode type.

| Nr | Age | Sex | Etiology of hearing loss | Age at onset HL | Side | Age at Impl. | Implant type |
| --- | --- | --- | --- | --- | --- | --- | --- |
| 1 | 45 | F | Cogan’s syndrome | 36 (2016) | left | 44 | CI622-SlimStraight-CP1110 |
| 2 | 45 | F | Sjögren’s syndrome | 36 (2016) | left | 38 | CI522-SlimStraight-CP1000 |
| 3 | 75 | F | GPA | 62 (2012) | left | 69 | CI622-SlimStraight-CP1000 |
| 4 | 75 | F | Microscopic polyangiitis | 55 (2005) | left | 65 | CI522-SlimStraight-Kanso |
| 5 | 72 | F | RA | 57 (2010) | right | 70 | Synchrony2-Flex28-Rondo3 |
| 6 | 76 | M | RA | 41 (1990) | right | 73 | Synchrony2-Flex28-Rondo3 |
| 7 | 76 | M | RA | 52 (2003) | left | 70 | Synchrony2-Flex28-Sonnet2 |
| 8 | 45 | F | sudden unilateral SNHL | 10 | left | 37 | CI522-SlimStraight-CP1000 |
| 9 | 43 | F | progressive bilateral SNHL | 15 | right | 39 | CI622-SlimStraight-CP1000 |
| 10 | 52 | F | progressive bilateral SNHL | 5 | left | 49 | CI622-SlimStraight-CP1000 |
| 11 | 39 | F | otosclerosis, bilateral progressive HL | 26 | left | 37 | CI622-SlimStraight-Kanso2 |
| 12 | 47 | F | sudden unilateral SNHL |  | right | 38 | CI522-SlimStraight-Kanso |
| 13 | 39 | F | progressive bilateral SNHL | 5 | right | 30 | Synchrony2-Flex28-Sonnet  Upgrade Sonnet2 2021 |
| 14 | 71 | F | sudden unilateral profound SNHL, mild progressive SNHL contralateral | 62 | left | 68 | CI622-SlimStraight-Kanso2 |
| 15 | 71 | F | progressive bilateral SNHL/MM | 60 | left | 63 | CI522-SlimStraight-Kanso  Upgrade Kanso2 2024 |
| 16 | 78 | F | progressive bilateral SNHL | 5 | left | 72 | CI522-SlimStraight-Kanso |
| 17 | 73 | F | progressive bilateral SNHL | 60 | right | 70 | CI622-SlimStraight-Kanso2 |
| 18 | 73 | F | progressive bilateral SNHL | 49 | left | 70 | Synchrony2-Flex28-Rondo3 |
| 19 | 81 | F | progressive bilateral SNHL | 66 | right | 75 | CI522-SlimStraight-CP1000 |
| 20 | 70 | F | progressive bilateral SNHL/MM | 57 | right | 67 | Synchrony2-Flex28-Rondo3 |
| 21 | 72 | F | progressive bilateral SNHL | 55 | left | 70 | Synchrony2-Flex28-Rondo3 |
| 22 | 78 | F | progressive bilateral SNHL | 63 | right | 73 | Synchrony2-Flex28-Rondo3 |
| 23 | 76 | M | sudden unilateral SNHL/moderate progressive SNHL contralateral | 56 | right | 71 | Synchrony2-Flex28-Rondo3 |
| 24 | 74 | M | sudden unilateral SNHL | 63 | right | 64 | Synchrony-Flex28-Sonnet upgrade Sonnet3 2025 |
| 25 | 79 | M | recurrent bilateral SNHL, possible noise trauma | 63 | right | 73 | Synchrony-Flex26-Rondo2  Upgrade Rondo3 2024 |
| 26 | 72 | M | progressive bilateral SNHL | 52 | left | 61 | Synchrony2-Flex28-Sonnet2 |
| 27 | 66 | M | sudden unilateral SNHL | 51 | left | 58 | Concerto-Flex28-Sonnet  Upgrade Sonnet2 2023 |
| 28 | 71 | M | sudden recurrent unilateral SNHL, mild progressive SNHL contralateral | 63 | right | 67 | Synchrony2-Flex28-Rondo3 |

Table S2: detailed clinical data of the study group, including autoimmune disease, serological testing and organ involvement as well as treatment details.

| Patient | Autoimmune disease | Serological testing | Organ manifestations | Treatment |  |
| --- | --- | --- | --- | --- | --- |
| 1 | Cogan’ syndrome | CRP, ESR  elevated | interstitial keratitis, sicca-symptoms, Raynaud’s syndrome, acute aortitis, SNHL | Methotrexate 15 mg s.c., Prednisolone 25 mg, Infliximab i.v. | |
| 2 | Sjögren’s syndrome | S-ANA positive | xerostomia, keratoconjunctivitis, arthritis, SNHL | Prednisolone 5 mg | |
| 3 | granulomatosis with polyangiitis (GPA) | c-ANCA positive, proteinase 3 ANCA | chronic otitis media, recurrent sinusitis, renal involvement, SNHL | Prednisolone 5mg, Rituximab i.v. | |
| 4 | microscopic polyangiitis (MPA) | p-ANCA, anti-ds DNA and myeloperoxidase positive | interstitial lung disease, Raynaud’s syndrome, antiphospholipid syndrome, SNHL | Prednisolone 5 mg, Azathioprine 50 mg, | |
| 5 | rheumatoid arthritis (RA) | anti-CCP antibodies, rheumatoid factor increased | arthritis, synovitis, SNHL | Methotrexate 15mg s.c., Etanercept 25mg s.c, + Leflunomide 10 mg  Methotrexate + Adalimumab s.c. | |
| 6 | RA | seronegative,  S-ANA increased | arthritis, synovitis, SNHL | Prednisolone high dose,  Methotrexate 15mg s.c, | |
| 7 | RA | anti-CCP antibodies, rheumatoid factor increased | arthritis, renal involvement, SNHL | Leflunomide 10 mg | |

**Medical history of study cohort**

In the study cohort one patient was diagnosed with Cogan’s syndrome, a systemic vasculitis affecting mixed-calibre vessels. In addition to audiological symptoms with fluctuating hearing, tinnitus, and vertigo, the patient showed signs of interstitial keratitis, laboratory tests revealed an elevated CRP and an increased erythrocyte sedimentation rate (ESR). Long-term therapy included systemic prednisolone, intensified with methotrexate, and escalated to infliximab (monoclonal TNFα-antibody) as progression with acute aortitis was diagnosed. A second patient presented with sudden progressive hearing loss and vertigo, due to ocular dryness he was diagnosed with Sjögrens syndrome, showing elevated serum anti-nuclear antibody (ANA) titers and positive Saxon’s and Schirmer’s tests. Sjögrens syndrome is primarily affecting the salivary glands, causing sicca symptoms, but is also associated with vasculitis and polyneuropathy. The patient received a long-term therapy with low-dose systemic prednisolone.

Among the cohort, two patients had a prior diagnosis of ANCA (antineutrophil cytoplasmatic antibodies)-associated vasculitis, comprising one case of GPA and one of microscopic polyangiitis (MPA). GPA was confirmed based on elevated c-ANCA levels, and the patient had a history of recurrent otitis media with unilateral acute hearing loss during a vasculitis flare. Following initial low-dose prednisolone therapy, the patient with GPA was escalated to rituximab (monoclonal anti CD-20-antibody) upon progression of pulmonary disease including tracheobronchial inflammation with bronchial stenosis. The patient with MPA was diagnosed based on elevated p-ANCA, myeloperoxidase and anti-ds DNA titers in the presence of arthritis and Raynaud’s syndrome and pulmonal involvement with ground-glass opacities and reduced lung function. This patient developed progressive hearing loss with tinnitus and vertigo and was treated initially with prednisolone, followed by azathioprine.

Three patients were diagnosed with rheumatoid arthritis (RA). The first patient developed profound hearing loss accompanied by tinnitus, fluctuation and aural fullness, but did not experience vertigo. Subsequently he was diagnosed with seropositive RA, confirmed by the presence of rheumatoid factor and positive anti-cyclic citrullinated peptide (anti-CCP) antibodies, and presented with symptoms of arthritis and synovitis. Initial treatment with methotrexate, followed by leflunomide, proved ineffective and therapy was escalated to the TNFα-inhibitor etanercept. This was later changed to a combination of methotrexate and adalimumab (monoclonal TNFα-antibody).

The second patient developed bilateral progressive hearing loss without associated tinnitus or vertigo. This patient was diagnosed with seronegative RA based on MRI findings demonstrating arthritis and synovitis of the hand. The third patient presented with progressive hearing loss characterized by fluctuations in hearing and episodes of vertigo. Seropositive RA was later diagnosed, and treatment with leflunomide (10 mg daily) was initiated. Interestingly, both patients with seropositive RA experienced tinnitus and vertigo in addition to fluctuating and progressive hearing loss, whereas the seronegative RA patient did not report any vestibular symptoms. In all three patients, hearing loss occurred up to 10 years before the diagnosis of RA was established, but it was never clinically attributed to the disease.

**Treatment of autoimmune disorders – potential for ototoxicity?**

Table S3: summary of data on drug-induced ototoxicity of immunosuppressant and immunomodulatory monoclonal antibodies and references to cases discussing cases of hearing loss after treatment

| Drug | pharmacological mechanism | suggested mechanism of ototoxicity | corresponding studies |
| --- | --- | --- | --- |
| Methotrexate  *antimetabolite* | Inhibiting cell replication by acting as analogues of compounds essential for DNA synthesis | neurotoxicity | - intratympanic injection in rats showed no ototoxicity^1^  - in pediatric patients 37 % of MTX treated patients later developed mild to moderate HL^2^ |
| Azathioprine  purine synthesis inhibitor | Interferes with purine synthesis which is essential for DNA and RNA | neurotoxicity | - ototoxicity reported in 4 patients with RA ^3^  - HL in a patient after renal transplantation.^4^ |
| TNF-α inhibitors  Etanercept  Infliximab  Adalimumab | inhibiting inflammatory cascade, reducing cytokine release and immune cell activation | drug-induced autoimmune vasculitis | - 43% HL in patients with ankylosing spondylitis treated with ^5^  - HL in 2 patients treated with adalimumab^6^  - HL reported in Italian drug database upon TNF-α inhibitor treatment^7^  - study reported no ototoxicity in 22 patients with RA^8^ |
| Rituximab  monoclonal CD20-antibody | antibody against CD-20 surface protein leading to depletion of B-cells | unclear | - report of HL in French drug database after treatment with monoclonal antibody^9^  - one patient with HL after rituximab therapy^10^ |
| Leflunomide  inhibitor of dihydroorotate dehydrogenase | inhibition of pyrimidine synthesis, affecting T- and B-cell activation | unclear | - mention of adverse events/HL in French drug database, no further case reports^9^ |

Recent reviews have addressed potential drug-induced ototoxicity from different therapeutic perspectives: Arya et al. (2024) examined monoclonal antibodies, while Franz et al. (2022) reviewed immunosuppressive drugs and their potential auditory adverse effects.^11,12^ Both studies highlight that comparison of reported ototoxicity cases is challenging due to several methodological limitations, including inconsistent or missing data on treatment duration, predominantly retrospective study designs, heterogeneous management strategies ranging from dose adjustment to drug discontinuation, and non-standardized follow-up procedures. Overall, calcineurin inhibitors are the immunosuppressants most commonly associated with ototoxic effects, typically related to elevated serum drug levels. None of our study patients received treatment with calcineurin-inhibitors. In contrast, ototoxicity linked to other immunosuppressive agents is rare and largely based on isolated reports, and the underlying mechanisms remain speculative. Consequently, definitive conclusions regarding immunosuppressant-induced ototoxicity remain limited.

**References ototoxicity**

1.	Eren SB, Dogan R, Yenigun A, et al. Evaluation of ototoxicity of intratympanic administration of Methotrexate in rats. *Int J Pediatr Otorhinolaryngol*. 2017;100:132-136. doi:10.1016/j.ijporl.2017.06.035

2.	Moore B, Sheets G, Doss J, et al. Is Methotrexate Ototoxic? Investigating the Ototoxic Late Effects of Pediatric Cancer Treatment. *Am J Audiol*. 2023;32(3):657-664. doi:10.1044/2023_AJA-22-00157

3.	Ahmadzadeh A, Daraei M, Jalessi M, et al. Hearing status in patients with rheumatoid arthritis. *J Laryngol Otol*. 2017;131(10):895-899. doi:10.1017/S0022215117001670

4.	Jenkinson PW, Syed MI, Mcclymont L. Progressive, reversible sensorineural hearing loss caused by azathioprine. *J Laryngol Otol*. 2014;128(9):838-840. doi:10.1017/S0022215114001807

5.	Savastano M, Marioni G, Giacomelli L, Ramonda R, Ferraro SM, Punzi L. Sensorineural hearing loss in ankylosing spondylitis treated with TNF blockers. *B-ENT*. 2010;6(3):183-188.

6.	Conway R, Khan S, Foley-Nolan D. Use of adalimumab in treatment of autoimmune sensorineural hearing loss: a word of caution. *J Rheumatol*. 2011;38(1):176; author reply 176. doi:10.3899/jrheum.100593

7.	Barbieri MA, Cicala G, Cutroneo PM, et al. Ototoxic Adverse Drug Reactions: A Disproportionality Analysis Using the Italian Spontaneous Reporting Database. *Front Pharmacol*. 2019;10:1161. doi:10.3389/fphar.2019.01161

8.	Toktas H, Okur E, Dundar U, Dikici A, Kahveci OK. Infliximab has no apparent effect in the inner ear hearing function of patients with rheumatoid arthritis and ankylosing spondylitis. *Clin Rheumatol*. 2014;33(10):1481-1487. doi:10.1007/s10067-014-2625-z

9.	Drug‐induced hearing loss: a case/non‐case study in the French pharmacovigilance database - Favrelière - 2020 - Fundamental & Clinical Pharmacology - Wiley Online Library. Accessed February 4, 2026. https://onlinelibrary.wiley.com/doi/10.1111/fcp.12533

10.	Healy EG, Phadke R, Kidd M, Reilly MM, Lunn MP. Clinical, neuropathological and radiological evidence for a rare complication of rituximab therapy. *Neuromuscul Disord*. 2015;25(7):589-592. doi:10.1016/j.nmd.2015.04.004

11.	Arya P, Salmerón Y, Quimby AE, Wong K, Brant JA, Hwa TP. The Impact of Monoclonal Antibody Usage on Hearing Outcomes: A Systematic Review. *Laryngoscope*. 2025;135(2):491-506. doi:10.1002/lary.31763

12.	Franz L, Frosolini A, Parrino D, Lovato A, de Filippis C, Marioni G. Ototoxicity of Immunosuppressant Drugs: A Systematic Review. *J Int Adv Otol*. 2022;18(2):167-176. doi:10.5152/iao.2022.21416

13.	Favrelière S, Delaunay P, Lebreton JP, et al. Drug-induced hearing loss: a case/non-case study in the French pharmacovigilance database. *Fundamental & Clinical Pharmacology*. 2020;34(3):397-407. doi:10.1111/fcp.12533

Figure S1: Scattergrams according to the American Academy of Otolaryngology – Head and Neck Surgery minimal reporting standard for reporting audiometric data. Generated using the software “R” (version 4.5.2, R Foundation for Statistical Computing, Vienna, Austria) and the R package from the following publication:
Pan DW, Oghalai JS. The AudioScatter Package in RStudio to Generate Scattergrams for Reporting Hearing Results. *Otol Neurotol*. 2026;47(1):7-10. doi:10.1097/MAO.0000000000004693
